# Derivation of a frailty index from the resident assessment instrument – home care adapted for Switzerland: a study based on retrospective data analysis

**DOI:** 10.1186/s12877-017-0604-3

**Published:** 2017-09-07

**Authors:** Catherine Ludwig, Catherine Busnel

**Affiliations:** 1 University of Applied Sciences and Arts of Western Switzerland, School of Health Sciences – Geneva, Avenue de Champel 47, 1206 Geneva, Switzerland; 2 Geneva Institution for Homecare and Assistance (imad), Avenue du Cardinal Mermillod 36, 1227 Carouge, Switzerland

**Keywords:** Frailty, Home care, Resident assessment instrument, Aging

## Abstract

**Background:**

The screening of frail individuals at risk for functional health decline and adverse health outcomes lies in the evolving agenda of home care providers. Such a screening can be based on a frailty index (FI) derived from data collected with interRAI instruments used in clinical routines to define care plans. The objective of this study was to assess the feasibility of deriving an FI from the Resident Assessment Instrument – Home Care adapted for Switzerland (Swiss RAI-HC).

**Methods:**

Data were collected by the Geneva Institution for Homecare and Assistance in clinical routines. The sample consisted of 3714 individuals aged 65 or older (67.7% females) who had each received a Swiss RAI-HC upon admission in the year of 2015. The FI was derived from 52 variables identified and scored according to published guidelines. Adverse health outcomes were either assessed during follow-up assessments (falls, hospitalizations) or documented from administrative records (mortality).

**Results:**

The results showed that the FI was distributed normally, with a mean of 0.24 (± 0.13), an interquartile range of 0.16, and values of 0.04 at percentile 1 and 0.63 at percentile 99. The effect of Age was significant (R^2^ = 0.011) with a slope of β = 0.002, 95% CI = [0.001–0.002]. Sex as well as the Age × Sex interaction were not significant. The FI predicted deaths (OR = 9.99, 95% CI = [3.20–29.99]), hospitalizations (OR = 3.40, 95% CI = [1.78–6.32]), and falls (OR = 5.00, 95% CI = [2.68–9.38]).

**Conclusions:**

The results support the feasibility of an FI derivation from the Swiss RAI-HC, hence replicating previous demonstrations based on interRAI instruments. The results also replicated findings showing that the FI is a good predictor of adverse health outcomes. Yet, the results suggest that home care recipients demonstrate a frailty pattern different from the one reported in community dwellers but comparable to clinical samples. Further work is needed to assess the characteristics of the proposed index in community-dwelling, non-clinical samples for comparability with the existing literature and external validation

**Trial registration:**

ClinicalTrials.gov NCT03139162. Retrospectively registered May 2, 2017.

**Electronic supplementary material:**

The online version of this article (10.1186/s12877-017-0604-3) contains supplementary material, which is available to authorized users.

## Background

Developed countries are witnessing demographic and epidemiologic transitions characterized by population aging [1] and increasing rates of chronic diseases and comorbidities [2]. In this evolving context, the “contemporary” patient is often older than 75, is frequently highly multimorbid, and has high risks of functional decline [3]. His or her particular needs place new challenges on health systems in terms of clinical management [2, 3]. In response to these challenges, efforts are being made to encourage patient-centered coordinated and integrated care with reduced barriers between hospital and community care [3]. The “ambulatory switch” is therefore fostered not only to enhance patients’ quality of life and satisfaction but also to reduce health-related costs [4]. In the face of an overhauled health care system and the evolving health trajectories of aging individuals, institutions delivering home care are critical actors [4]. Indeed, along with demographic and epidemiologic transitions, the number of elderly persons benefiting from home care has been continuously rising [5, 6]. In Switzerland, 1.5% of the population aged 65 to 69 living in private homes benefit from home care, a rate that increases with age reaching 34% among elders aged 85 and above [6]. Parallel to the age-related increase in home care needs is the progressive loss of independence in activities of daily living attributed to declining health [1, 6, 7]. In Switzerland, 26% of the population aged 65 to 69 declare themselves to be moderately to severely dependent in activities of daily living, a rate that reaches 61% among elders aged 85 or older [6]. These rates, which are similar to those reported in Europe [7] and in most developed countries [1], suggest that the appropriate clinical management of the “contemporary” patient should include the prevention, detection, and management of functional decline [2] above and beyond specific disease management [3]. In this agenda, home care services have a definite role to play in the screening of individuals who are at risk of independence loss.

Frailty is among the health conditions or syndromes known to drastically increase the risk of functional loss [1]. Consensually, frailty is defined as a “multidimensional syndrome characterized by decreased reserve and diminished resistance to stressors. […] frailty represents a state of extreme vulnerability, where minimal stress may cause functional impairment” ([8], p.65–66). Frailty is also often recognized as a reversible state, especially in its early stages [9–11], which encourages early screenings when seeking prevention.

Many models have been proposed to operationalize frailty [8, 12]; among them is the model of frailty as an accumulation of deficits [13], in which frailty is scored by means of a frailty index (FI, [14]). The FI is computed as the number of deficits reported on a wide range of health conditions and diseases (ideally over 30, [15]), and the resulting ratio provides an estimate of the whole health of an individual, which can also stand as a proxy measure of aging [16]. By definition, the FI is not characterized by any underlying factorial structure or predefined subcomponents. An FI can be derived from various types of databases as long as its derivation follows the proposed guideline [15]. Recent studies convincingly demonstrated that an FI can be derived from data collected with unified instruments from the interRAI suite either with the interRAI Acute Care [17], interRAI Nursing Homes [18], or interRAI Home Care [19]. Aside from FI derivations, scales assessing frailty have also been proposed; among them, the Frailty Scale [20] built from the interRAI Home Care and the FRAIL-NH scale [21, 22] elaborated on the interRAI Nursing Home. The former operationalizes frailty as an accumulation of deficit [13], and the latter relies on the phenotype model of frailty [23], which assigns individuals to “robust”, “pre-frail”, and “frail” categories based on a restricted number of variables assessing predefined physiological resources. These two distinct ways of operationalizing frailty have common construct validity [24] but serve different purposes [25]. As the original phenotype operationalization of frailty, the FRAIL-NH is a useful classification tool, but as a categorical score, it holds weaker inter-individual discriminative power than the FI. Oppositely, the FI is finer grained and more sensitive to modifications, including inter-and intra-individual ones. The FI also appears to be best predictor of adverse health events [24], such as falls [26, 27], hospitalizations [26], and death [17, 19, 28–30].

In Switzerland, the Resident Assessment Instrument – Home Care adapted for Switzerland (Swiss RAI-HC) has been advised for more than a decade for defining care plans for every adult requesting home care. Accordingly, this instrument is used by the Geneva Institution for Homecare and Assistance (Institution genevoise de maintien à domicile, or “imad”) upon admission and for follow-ups with the primary purpose of defining adapted home care plans based on individual needs. With more than 3000 initial assessments per year, the available data offer a great opportunity to derive an FI from the Swiss RAI-HC data as previously done using interRAI instruments [17, 19]. We preferred the continuous FI over a categorical score to assess frailty, because the FI is more sensitive to inter-individual differences, and thus it better estimates frailty in various subsamples of the aged population [31]. It is also a better predictor of adverse outcomes [24]. Thus, the objectives of the present study were 1) to identify Swiss RAI-HC candidate variables in the Swiss RAI-HC that fulfill the requirements for FI derivation [15]; 2) to describe the characteristics of the FI distribution and the effects of Age and Sex on the FI values; and 3) to assess the predictive power of the FI on adverse health outcomes, specifically falls, hospitalizations, and deaths. In achieving these aims, we expect 1) to demonstrate that an FI can be derived from the Swiss RAI-HC as done previously with various interRAI instruments [17–19] and b) to provide evidence in favor of a good predictive validity of the proposed FI, and thus, replicate previous findings [17, 19, 24, 26–30].

## Method

### Data collection and preparation

The study was conducted on a dataset collected by imad using the Swiss RAI-HC [32] during the years 2015 and 2016. As the interRAI Home Care instrument [33], the Swiss RAI-HC entails a minimal dataset (MDS) covering a large panel of health-related domains. More specifically, it entails 147 items for 18 domains (see Additional file 1: Table S1). The assessments were done by trained clinical nurses as routine valuations upon either admission, reassessment purposes such as regular follow-ups (between three and nine months), or in the case of major changes in health conditions. The data collection was computer assisted using the MedLink® solution (Medical Link Services SA, Nyon, Switzerland), which automatically fed the database after each evaluation. The raw data were extracted from the database by imad and coded for the research team, that is, with single numeric identifiers for each participant but without any information allowing for personal identification. The original file was in Microsoft Excel format (Microsoft Corp., Redmond, WA, USA) secured by a password. Data were subsequently imported in SPSS 22.0 for Windows (IBM Corp., Armonk, NY, USA) for variable recoding and analysis purposes.

### Study sample

The study sample was drawn from the 2015–2016 database, which included 11,888 records, each one corresponding to a full assessment done with the Swiss RAI-HC. Among these records, only those corresponding to men and women aged 65 or older at the time of the assessments were considered (*N* = 10,384, i.e. 87.35% of the available data). For each home care recipient, the first assessment upon admission done in 2015 was considered (*N* = 3839), whereas reassessments (*N* = 6191) and corrections (*N* = 354) were dropped. Finally, participants with missing data (*N* = 125) related to the variables used to compute the FI were excluded, leaving a study sample of 3714 individuals (67.7% females) aged on average 82.7 ± 7.7 (M ± sd) years. This initial sample was monitored until the end of 2016. Over this period, one (*N* = 2816) to eight (*N* = 18) follow-up examinations were recorded either as regular reassessments or due to significant changes in health conditions. Follow-up data were used to identify individuals who fell and/or were hospitalized after the initial Swiss RAI-HC assessments.

### Derivation of the frailty index (FI)

The FI was derived from the rationale used in published studies [17–19], and in accordance with the proposed guidelines [14], which recommend 1) the number of considered variables be greater than 30 and that these variables 2) be associated with health status. Further the variables have 3) to reflect a variety of physiological systems/deficits; 4) to assess outcomes or deficits with a documented age-related increase in prevalence; and 5) to avoid floor or ceiling effects. In this study, the FI was derived from a set of 52 variables identified among the 147 variables available in the Swiss RAI- HC MDS. Variables were selected using a Delphi consensus building approach [34] involving two experts (one in gerontology, the second in homecare nursing) who first addressed each of the 147 items of the Swiss RAI-HC with criteria 2 and 4. A second round was conducted to address criteria 1 and 3, and a descriptive analysis was conducted on the candidate item to ensure that criterion 5 was fulfilled. The 52 items consensually selected covered a variety of systems fulfilling criterion 3 including attention, memory, language, orientation, emotion and affect, sensory abilities, functional health, nutrition, medication, physiology, and pain (see Additional file 2: Table S2). Each item was recoded so as to score the absence/presence of a deficit (see Additional file 3: Table S3). For variables originally coded “0 = absence” and “1 = presence” of a health problem, the presence and absence of deficits were recoded accordingly. For health problems coded as either “absent = 0” or present with various gradations of deficits, coding identified deficits as either “absent = 0” or as “present = 1”, irrespective of the gradation. For items in which the original response scale entailed the modality of “respondent does not answer,” this modality was further coded as “1 = deficit”, as Hubbard et al. previously did [18]. For items in which a given problem was coded “absent = 0”, “present but newly observed = 1”, or “present but not new = 2”, recoding was “0 = deficit absent” and “1 = deficit present”, be it newly observed or not. For sensory and communication abilities, incontinence, and dyspnea, which included the response modality of “most of the time” if the deficit was absent, items were recoded as deficit “absent = 0” if the deficit was totally absent, “deficit = 0.5” if the deficit was absent most of the time, and “deficit present = 1” for all other remaining cases. Functional health items were also recoded using a 0.5 value for all answers qualifying as activities conducted with help but not requiring strength. Finally, continuous original values such as the body mass index (BMI) and the number of different types of medication taken over the previous seven days were categorized. BMI was coded as “deficit = 1” if the index was <21 or ≥ 30, else the deficit was coded as “absent = 0”. As Hubbard et al. [17] proposed, medication was recoded as deficit “absent = 0” if the number of medications ranged from 0 to 2, “deficit = 1” if the number ranged from 3 to 8, and “deficit = 2” if the number was greater than 8. For each participant, the FI was computed as the sum of deficits reported for a given individual divided by the number of candidate variables (*N* = 52).

### Outcome variables

To assess the predictive value of the FI on health outcomes, the FI continuous ratio score computed from 52 variables was used as predictor, and three outcome variables were considered: falls, hospitalizations, and deaths. Falls and hospitalizations were documented by means of the Swiss RAI-HC follow-up assessments, respectively, with falls occurring fewer than 90 days prior to assessment (item K3), and hospitalizations occurring from 7 to 90 days prior to assessment (item A3). Across follow-up assessments, only the first occurrences of falls and/or hospitalizations were considered. Deaths were documented from administrative records and provided by imad. The occurrence of each outcome was documented as “present” or “absent” throughout the 1-year period covered in the dataset, without any time-to-event information; thus binary coding was used.

### Statistical analyses

Statistical analyses were carried out with SPSS 22.0 (IBM Corp., Armonk, NY, USA). First, descriptive statistics were conducted to compute the frequency of deficits for each candidate item considered to derive the FI. This analysis was done to assess possible floor or ceiling effects, which, according to the recommendations [15], should not be present. Second, descriptive statistics were conducted to describe the distribution of the FI. For each distribution parameter, 95% confidence interval (95% CI) estimates were additionally computed using 1000 bootstraps. Third, Cronbach Alphas were computed to assess the internal consistency of the IF. Values were estimated for the entire scale with the 52 items and for the scale with each of the items deleted. Inferential statistics were conducted to assess the Age and Sex effects on the derived the FI. Analyses were done using linear regressions with the FI as the outcome variable and with Age and Sex as predictors. An additional Age × Sex interaction term was used, and Wald statistics were applied. Confidence estimates of coefficients were computed using bootstrapping with 1000 bootstraps. Analyses assessing the predictive value of the FI were done using logistic regression models with binary outcomes for falls (0 = no fall, 1 = fall), hospitalizations (0 = not hospitalized, 1 = hospitalized), and deaths (0 = not deceased, 1 = deceased). In the absence of precise time-to-event information, logistic regressions appeared best suited to assess the likelihood of an outcome occurrence as a function of frailty score over the time period considered. For each of these outcomes, Age and Sex were entered as predictors. For each model, the 95% CI of coefficients were estimated using bootstrapping with 1000 bootstraps. A last set of analyses was completed using receiver operation curves (ROC) and the Youden Index (or J statistics) [35] computation to identify the FI optimum cut-off points in terms of the diagnostic accuracy of the FI with respect to mortality, hospitalizations, and falls. For this set of analyses, the dichotomous outcome variable was entered as a state variable, and the linear FI index was entered as a test variable. Areas under the curve (AUC) and associated *p*-values were estimated, and J-statistics were computed to identify FI cut-off points.

### Ethics

The trial is qualified as a retrospective study using coded data on non-genetic health personal data; it was retrospectively registered. Written consents for the use of individual Swiss RAI-HC data for research purposes and research result disseminations were obtained from each individual or from a proxy before the assessment. The protocol was approved by the Ethical Committee of Canton Geneva, Switzerland.

## Results

### Study sample

From the sample of 3839 individuals aged 65 or older who were assessed upon admission during the year of 2015, a total of 125 (3.3%) were excluded due to missing data on the variables used to compute the FI. The remaining sample consisted of 3714 individuals (67.7% females) aged, on average, 82.7 ± 7.7 (M ± sd) years. A *t*-test for independent samples revealed that women (M = 83.5, sd = 7.5) were significantly older than men (M = 81.2, sd = 7.8), with a mean difference of 2.3, 95% CI = [1.75–2.80], t (3712) = 8.54, *p* < 0.001, yet with a small to moderate significance as Cohen’s effect size value of d = 0.30 suggested. Given this significant age difference, all inferential analyses were conducted by taking age into account.

**Fig. 1:**
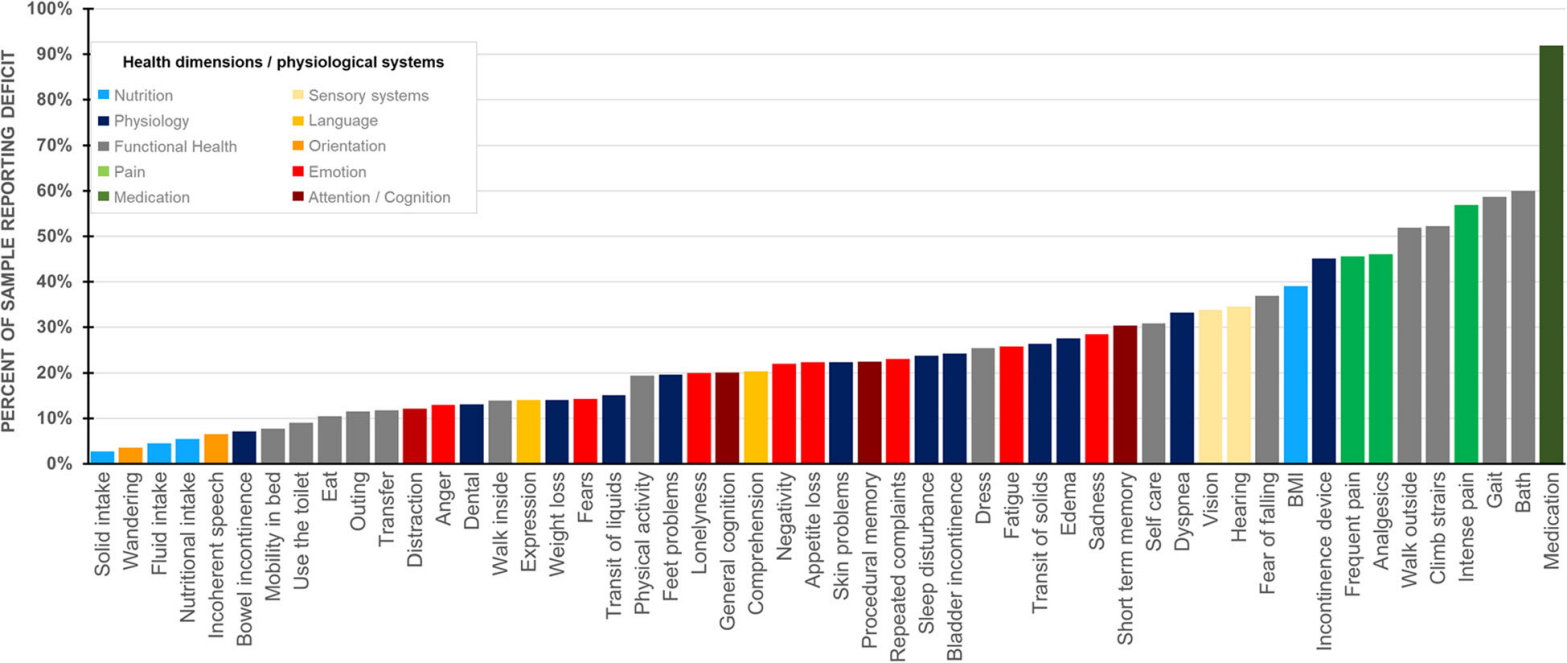
Frequency of deficit report by item for each of the 52 items considered for the FI. Health dimensions/physiological systems (*N* = 10) are color-coded

### Frailty index (FI)

The results of the descriptive analysis (see Fig. 1) conducted on each item for the estimation of the deficit frequency revealed that the frequency ranged from 2.7% (*N* = 101 individuals) for the item assessing solid intake to 91.9% (*N* = 3412) for medication, hence supporting the absence of floor or ceiling effects in the candidate items. The results of the descriptive analyses done to characterize the distribution of the FI revealed an average value of 0.24 [0.24–0.24] ± 0.13, (M [95% CI] ± sd,), ranging from 0.04 at percentile 1 to 0.63 at percentile 99, with an interquartile range of 0.16. Values were observed separately for men and women, respectively: 0.23 [0.23–0.24] ± 0.13 for men and 0.24 [0.24–0.25] ± 0.13 for women. In addition, values at percentiles 1 and 99 and interquartile ranges were identical as reported on the entire sample. All parameters had small 95% CI estimates, suggesting robust estimations. Finally, the internal consistency of the scale was good, revealed by a Cronbach Alpha of 0.847, ranging from 0.843 (when item H2e “walk outside”, was deleted) to 0.850 (when categorized BMI was deleted).

**Fig. 2:**
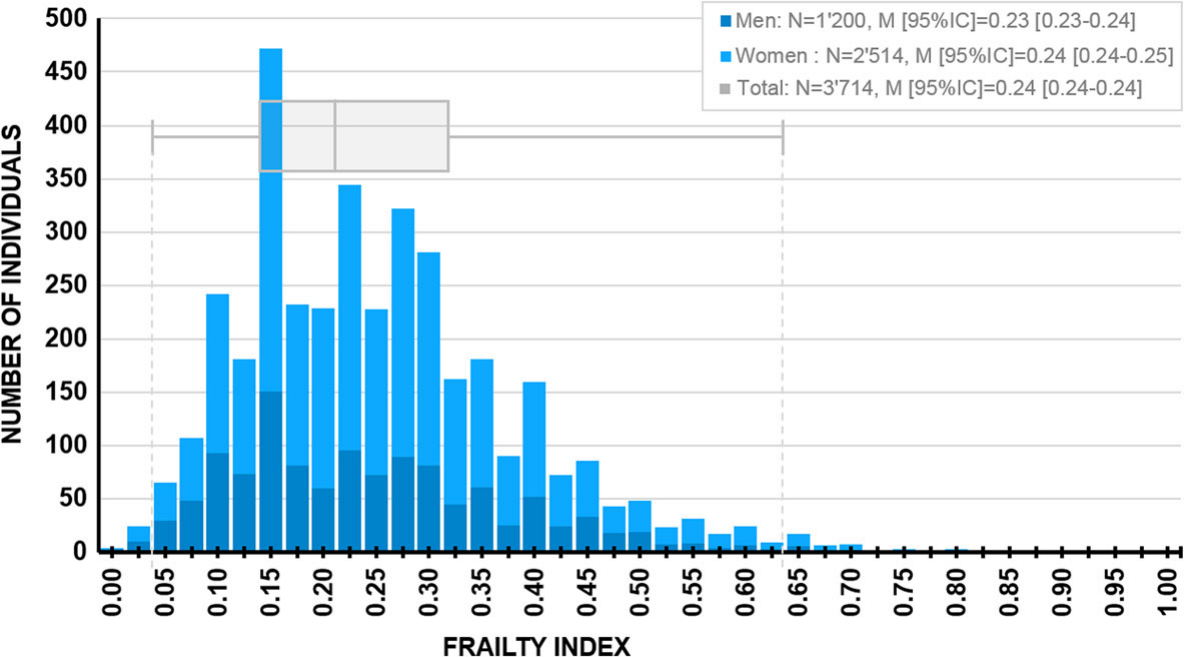
Distribution of the frailty index by Sex and for the entire sample. M: Mean; 95% CI: 95% Confidence intervals of the mean estimated by bootstrapping (*N* = 1000). The box-and-whisker plot represents the values for the total sample, including the median, the values at percentiles 25 and 75, and the values at percentiles 1 and 99 (extremes)

The detailed distribution of the frailty index by sex and for the entire sample is provided in Fig. 2. The results of the linear regression analysis assessing the effects of Age, Sex, and the Age × Sex interaction revealed that Age was significant, R^2^ = 0.011, Wald (1, 3710) = 35.33, *p* < 0. 001 with a slope of β = 0.002, 95% CI = [0.001–0.002]. On the contrary, Sex was not significant, Wald (1, 3710) = 0.001, *p* = 0.977, and neither was the Age × Sex interaction, Wald (1, 3710) = 0.03, *p* = 0.854. The overall effect of the model was significant, Wald (3, 3710) = 46.46, *p* < 0.001.

### Predictive value of the FI

Concerning mortality, 158 out of the 3714 (4.3%) individuals in the sample died during the follow-up period; among the 158, 78 were men and 80 were women. The age at FI assessment was 84.6 ± 8.0 years (M ± sd), and the age at death was 85.2 ± 8.0 years. For men, the corresponding years of age were 83.6 ± 7.7 and 84.1 ± 7.8; for women, they were 85.6 ± 8.2 and 86.2 ± 8.2. The interval between the examination and death was on average 12.56 ± 4.29 (M ± sd) months, ranging from 1.48 to 20.25 months.

Concerning falls and hospitalizations, the analyses were conducted on a subsample of 2816 individuals for whom reassessments with the Swiss RAI-HC were available (i.e., 75.8% of the initial sample). Among them, 1914 were women (68.0%) and 902 men (32.0%), respectively, aged on average 83.81 ± 7.40 (M ± sd) years and 81.58 ± 7.59 years at first examination.

With respect to falls, 1117 individuals (60.4%) fell at least once during the monitoring period, and among them, 774 (69.3%) were women. For hospitalizations, 1259 individuals (55.3%) were hospitalized at least once after the initial assessment, and among them, 835 (66.3%) were women. The results of descriptive statistics for the FI values by Type of adverse event and Sex are provided in Table 1. The results of the logistic regression analyses for mortality, hospitalizations, and falls are reported in Table 2.

**Table 1:** Descriptive statistics of the FI in presence or absence of adverse health events

	Adverse event				No adverse event			
	N	M-FI	95% CI	sd	N	M-FI	95% CI	sd
*Mortality*								
All	158	0.28	[0.26–0.31]	0.14	3556	0.24	[0.23–0.24]	0.13
Men	78	0.25	[0.22–0.28]	0.13	1122	0.23	[0.22–0.24]	0.13
Women	80	0.32	[0.28–0.35]	0.15	2434	0.24	[0.24–0.25]	0.12
*Hospitalizations*								
All	1259	0.25	[0.24–0.26]	0.12	1557	0.23	[0.23–0.24]	0.12
Men	424	0.25	[0.24–0.26]	0.13	478	0.22	[0.21–0.23]	0.12
Women	835	0.25	[0.24–0.26]	0.12	1079	0.24	[0.23–0.25]	0.12
*Falls*								
All	1117	0.26	[0.25–0.26]	0.12	1699	0.23	[0.23–0.24]	0.12
Men	343	0.26	[0.24–0.27]	0.12	559	0.21	[0.20–0.22]	0.12
Women	774	0.26	[0.25–0.27]	0.12	1140	0.24	[0.23–0.25]	0.12

*M-FI* Mean value of FI, *95% CI* 95% Confidence interval of the mean, *sd* Standard deviations. Values are estimated by bootstrapping (N = 1000)

**Table 2:** Results of logistic regressions assessing the effect of FI, age, and sex on adverse health outcomes

	OR	95% CI	Wald	*p*-value
*Falls* ^a^				
FI	5.00	[2.68–9.38]	26.12	< 0.001
Age	1.01	[1.00–1.03]	7.48	0.006
Sex^c^	0.96	[0.81–1.14]	0.28	0.598
*Hospitalizations* ^a^				
FI	3.40	[1.78–6.32]	15.53	< 0.001
Age	0.98	[0.97–0.99]	11.81	0.001
Sex^c^	1.13	[0.96–1.33]	2.06	0.152
*Death* ^b^				
FI	9.99	[3.20–29.99]	16.50	< 0.001
Age	1.04	[1.01–1.07]	11.27	0.001
Sex^c^	2.37	[1.72–3.36]	27.04	< 0.001

*OR* Odd ratio, *95% CI* 95% Confidence interval, *Wald* Wald Chi-square value. Values estimated by bootstrapping (*N* = 1000). ^a^Sample of 2816 individuals; ^b^Sample of 3714 individuals; ^c^Females used as reference

**Fig. 3:**
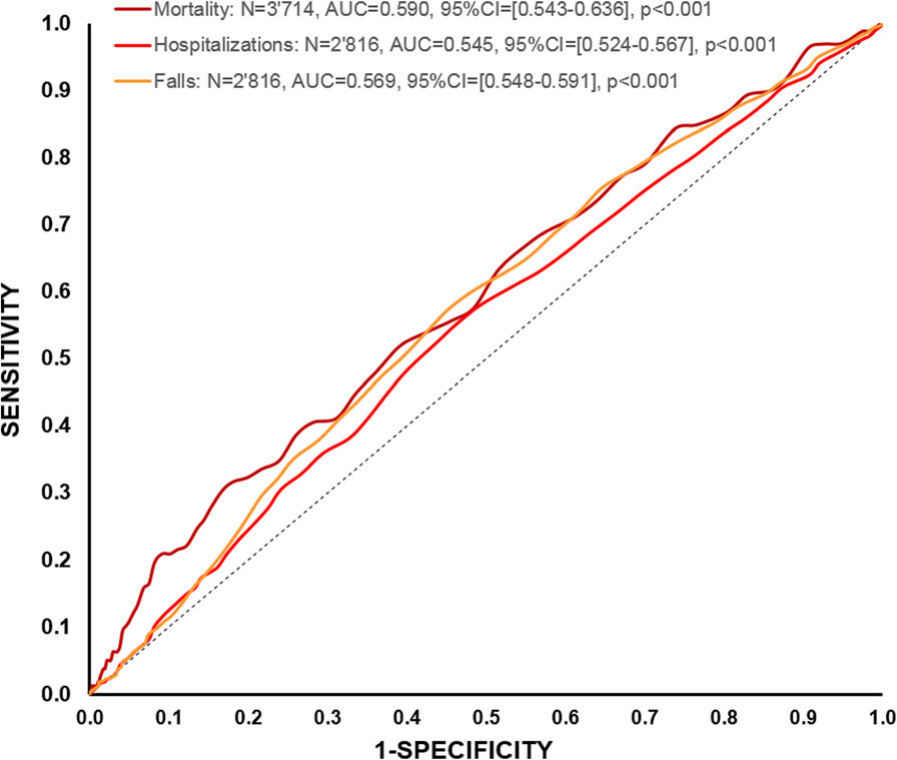
Receiver operating curves (ROC) for FI in relation to mortality, hospitalizations, and falls. Dashed line represents chance level. AUC = area under the curve. 95% CI = 95% confidence interval of AUC; *p* = *p*-values

Finally, the results of the ROC analyses are displayed in Fig. 3, illustrating the diagnostic accuracy estimation of FI with respect to mortality, hospitalizations, and falls. The results demonstrate that, albeit significant at the *p* < 0.001 level, the diagnostic accuracy of FI was modest [36] for mortality, AUC = 0.590, 95% CI = [0.543–0.636], hospitalizations, AUC = 0.540, 95% CI = [0.524–0.567] and falls AUC = 0.569, 95% CI = [0.548–0.591]. Youden indices of optimal criteria were J = 0.137 with FI = 0.35 for mortality, J = 0.089 with FI = 0.23 for hospitalizations, and J = 0.122 with F = 0.23 for falls.

## Discussion

The present study was aimed at deriving an FI from the Swiss RAI-HC as previously reported with respect to the interRAI Acute Care [17] and the interRAI Home Care [19]. The sample considered for the study consisted of individuals assessed upon admission for home care, aged on average 82.7 ± 7.7 (M ± sd), with the majority of them being women (67.7%), comparable to the sample that Armstrong et al. [19] studied in the home care setting (*N* = 23,952; age: 81.7 ± 7.4 years, 69.4% female) yet with slightly more women than in the sample that Hubbard et al. [18] studied (*N* = 1418, age: 81.0 ± 6.8, 55.0% female). However, these samples appear to fit the “contemporary patient” characteristics [3] at least in terms of age (> 75 years old) and sex (higher proportion of women).

Concerning the descriptive characteristics of the FI, the derived results reported a mean value of FI = 0.24 ± 0.13 (M ± sd), corresponding to an average of 11.44/52 deficits. This value falls into to the range previously reported in home care settings (FI = 0.18 to FI = 0.42; 9/50 to 21/50 deficits [19]), although it is slightly lower than the value reported in acute care (FI = 0.32 ± 0.14, 18/56 deficits [17]). The FI was normally distributed, and the 99% upper limit of the value did not reach the theoretical maximum (FI = 1); rather, it was 0.63 in the range of the maximum values (0.65 ± 0.05) previously documented [17, 19, 37]. The FI demonstrated a good internal consistency. In relation to age, our results revealed a significant, albeit very modest effect (β = 0.002) of age, replicating previous findings of a slope < 0.01 reported for clinical samples [13] or in acute care [17]. Meanwhile, in community dwelling samples, the relation between age and the FI is usually greater with an estimated annual increment of 3% of the FI value [29, 37]. Such a difference can be explained by the fact that, in clinical, acute, and long-term care, samples might be frailer than individuals living in the community without specific care needs [29]. Concerning sex differences on the FI, our findings do not replicate previous findings that report higher FI values for women as compared to men [29]. This discrepancy of results could be accounted for by the fact that we assessed the effects of Sex, using regression modeling and considering not only this variable but also age and the Age × Sex interaction. A linear regression model considering Sex as a unique predictor of the FI (women as a reference) was performed a posteriori for comparison purposes. The results revealed a significant effect of Sex, in favor of men (β = −0.011, 95% CI = [−0.021 - -0.002], *p* = 0.015), suggesting that in our sample, sex differences were not independent of Age. This result further supports that the FI values in clinical sample it higher that the ones reported in community-dwelling elders.

Finally, concerning the predictive value of the FI, the results clearly demonstrated that independently of Age and Sex, the FI is a strong predictor of hospitalizations, falls, and mortality. In our sample, each 0.1 increase of the FI increased the likelihood of death by nearly 10 (OR = 9.9, 95% CI = [3.20–29.99]), the likelihood of falls by five (OR = 5.0, 95% CI = [2.68–9.38]), and the likelihood of hospitalizations by more than three (OR = 3.4, 95% CI = [1.78–6.32]), replicating previous findings on the predictive value of the FI in terms of adverse health events [17, 19, 26–30]. Yet, the considerable size of the observed effects further supports the assumption that home care recipients aged 65 and older constitute a highly fragile population with very high risks in terms of undesirable health outcomes. It is also important to mention here that a posteriori analyses were done to assess pre-existing differences between the sample that received a single RAI-HC assessment and the follow-up sample for which predictive analyses on falls and hospitalizations were assessed. The results revealed that the two samples significantly differed on Age, with the follow-up sample slightly older (83.10 ± 7.53, M ± sd) than the non-returning sample (81.65 ± 8.00), F (1, 3712) = 24.351, *p* < 0.001. However, the two samples did not significantly differ on either Sex χ2 (2, 3714) = 0.520, *p* = 0.539 (retuning, 67.97% of women vs. 66.82% in non-returning) or on Frailty, F(1, 3712) = 0.854, *p* = 0.355 (non- returning: FI = 0.24 ± 0.14; returning FI = 0.24 ± 0.13). The latter result ascertains that the reported pattern of FI prediction on falls and hospitalizations is not overestimated due to pre-existing higher level of frailty in the sample considered for the analyses.

The assumption of substantial frailty in the sample receiving care is also supported by the results of the ROC curve analyses and Youden index computation. First of all, AUC values were significant (*p* < 0.001) for falls, hospitalizations, and deaths suggesting that the FI allows for the assessment of the adverse outcomes’ considered risks. Yet, the reported values were lower than those reported for community dwellers (UAC ≅ 0.70; [27, 28] and were very modest in terms of the effect size (< 0.60; [36]), calling for further investigations in long-term care clinical populations. The last set of analyses, meant to define the FI cut-off score for risks of adverse events, reported FI values identical to the median value of the FI distribution (i.e., FI = 0.23) for falls and hospitalizations, suggesting that for 50% of the sample, the risks of falls and hospitalizations become critical. As a reminder, 39.7% of the sample with reassessment fell, and 44.7% were hospitalized at least once in the follow-up period. In comparison, the estimated one-year fall rate in the Swiss population aged 65 or older is of 25.2% among community dwellers and of 38.9% among nursing home resident [38]. As concerns one-year hospitalization rates among community dwellers aged 65 or older, Swiss survey data report 18% hospital stays [39] and 12% admission for ambulatory treatment or for emergency [40]. In other words, our sample of elders benefiting from sustained home care shows noticeably high rates of adverse events.

Concerning risks of mortality, the reported FI cut-off value was 0.35, slightly higher than the value reported at percentile 75 (i.e., 0.31). This suggests that, for individuals with FI scores in the upper 25% of the distribution, risks of death were critical, which is in line with previous reports [28] showing that survival probability is drastically reduced for “moderately frail” (FI = 0.36 ± 0.09) and “severely frail” (FI = 0.43 ± 0.08) community dwellers.

### Study strengths and limitations

The present study demonstrates that an FI index can be derived from data collected with the Swiss RAI-HC, as was previously shown using interRAI Home Care [19] and interRAI Acute Care [17]. Deriving a frailty estimate directly from assessments done in clinical routines has undoubtedly significant benefits, not only in terms of the gain of assessment time but also, more importantly, in terms of the frailty description, screening, and prevention in a home care population that is increasingly aged and multimorbid. Applying frailty information to the description of the current home care population should allow both service providers and nurses working in the field to have more knowledge regarding care recipients and thus provide ever more appropriate care. Follow-up assessments of the FI would further help develop knowledge about the evolution of frailty over time in the target population. As previously mentioned in the literature [25], the continuous nature of the FI makes this score well-suited to identify small inter-individual differences within subsamples of the aged population ranging from community-dwellers in good health to clinical samples of heavily disabled individuals. In addition, the FI provides means to grasp even small intra-individual changes when measured on consecutive occasions. Thus, the FI stands as the measure of choice for both early detection of frailty and follow-up studies [25, 31]. However, the FI is hardly applicable in clinical routines as a ready-to-use and easy to interpret decision tool as opposed to categorical frailty scores such as Fried’s phenotype [23] or FRAIL-NH [22] scores. Finally, since the FI—as the FRAIL-NH—are derived from prior comprehensive geriatric assessments, these scores are inapplicable at first contact and require some processing before use. Overall, the two categories of frailty instruments provide clinically distinct information and thus, serve different purposes. Accordingly, their combined use has been advised [25]. In future developments of the present study, the proposed FI could be tested against the a categorical score also derived from the MDS either based on Fried’s [23] phenotype or computed following the FRAIL-HN procedure [22]. This would allow to address the construct validity of the proposed IF. In addition, deriving categorical scores could also offer the opportunity to test different cut-off values and assess their predictive power in various adverse outcomes as done by Luo et al. [21].

Beyond the convincing evidence supporting the feasibility of deriving an FI from the Swiss RAI-HC, our results provide evidence that the population studied displays characteristics closer to those reported in clinical populations than to those reported among community dwellers. Yet, in terms of FI validity assessment and population characterization, further work applying the proposed methodology to non-clinical samples would be needed. Another limitation of the present study is its retrospective nature and the constraint imposed by the characteristics of data collected prior designing the analyses. An example is the partial record of adverse outcomes identified by odds but lacking time-to-event information. Yet, special care was placed to sequence measurement occasions to ensure that the FI score, used as a predictor, was derived from assessments conducted prior the occurrence of any considered outcomes. A prospective cohort study with documented dates for adverse outcomes would most likely bring a more precise estimation of risks through survival analyses, as well as more appropriate means to apprehend the evolution of the FI over time among home care recipients. Additional work would also be needed to enlarge the panel of health outcomes considered for a more detailed description of the predictive power of frailty and its implication in defining increasingly complex health trajectories. Further, reasons underlying hospitalizations should be recorded with care so as to distinguish planned and unexpected admissions, as well as admission due—or not due—to life threatening conditions. Such a detailed description would permit a more precise identification of hospital admissions that could be attributed to frailty, and to distinguish them from admissions that are not significantly accounted for by a general loss of resources. In the present study, as in many others, hospitalizations were considered irrespective of their underlying causes. As a result, the reported predictive value of frailty is probably overestimated and should be taken with some caution.

## Conclusion

In the context of demographic and epidemiologic transitions that industrialized countries witness, and in the face of the “ambulatory switch,” home care services are increasingly involved in the complex health trajectories of aged and multimorbid individuals in need of long-term and sustained yet individualized care. In this challenging context, the early screening and prevention of individuals at risk for functional loss are of growing concern. Today more than ever, frailty is a public health concern. The results of the present study bring additional pieces of evidence in supporting the feasibility and interest of deriving a frailty score from instruments that are used in clinical routines such as the Swiss RAI-HC. Although further work is still needed to recommend an algorithm for FI computation with effective applied properties in clinical settings, the proposed methodology appears suitable in identifying home care recipients that could benefit from proactive interventions aimed at reducing risks of adverse health outcomes.

## Additional files

Additional file 1: Table S1. The 18 sections of Swiss RAI-HC MDS and their corresponding content**.** This supplementary information lists the 18 sections entailed Minimum Data Set of the Swiss RAI-HC, and reports a brief description of their corresponding content. (DOCX 19 kb)

Additional file 2: Table S2. Set of items selected in the Swiss RAI-HC MDS to derive the FI**.** This supplementary information provides the set of items selected in the Swiss RAI-HC MDS to derive the FI. For each item, the information includes the original code from the reference manual, the health domain documented and the specific outcome assessed. (DOCX 19 kb)

Additional file 3: Table S3. Coding applied to the items selected in the Swiss RAI-HC MDS to derive the FI. This supplementary information reports the detailed coding applied to each of the items selected in the Swiss RAI-HC MDS to derive the FI. (DOCX 19 kb)

## Abbreviations

FI: Frailty index
imad: Geneva Institution for Homecare and Assistance [Institution genevoise de maintien à domicile]
LIPAD: Public information, document access and personal data protection Act of Canton Geneva, Switzerland [Loi cantonale genevoise sur l’information du public, l’accès aux documents et la protection des données personnelles (LIPAD)]
MDS: Minimum Data Set
RAI: Resident Assessment Instrument
RAI-HC: Resident Assessment Instrument – Home Care
