# Supplementary material for: Derivation of a frailty index from the resident assessment instrument – home care adapted for Switzerland: a study based on retrospective data analysis
Source: BMC Geriatr. 2017 Sep 7;17:205. doi: 10.1186/s12877-017-0604-3 (PMC5590146; doi:10.1186/s12877-017-0604-3)
Supplement: Supplementary file 1 — The 18 sections of Swiss RAI-HC MDS and their corresponding content. This supplementary information lists the 18 sections entailed Minimum Data Set of the Swiss RAI-HC, and reports a brief description of their corresponding content. (DOCX 19 kb) [file 12877_2017_604_MOESM1_ESM.docx]

**Additional file 1: Table S1.** The 18 sections of Swiss RAI-HC MDS and their corresponding content

| **Section / Number** | **Content documented** |
| --- | --- |
| A / 1 | Date of assessment |
| B / 2 | Cognitive status |
| C / 3 | Communication and hearing |
| D / 4 | Vision |
| E / 5 | Mood and behavior |
| F / 6 | Social functioning |
| G / 7 | Unformal care |
| H / 8 | Physical and functional functioning |
| I / 9 | Continence |
| J / 10 | Medical diagnoses (documented from available record information) |
| K / 11 | Health problems (clinical symptoms of digestive, respiratory, vascular, system dysfunction; falls, pain) |
| L / 12 | Nutritional state |
| M / 13 | Skin and feet problems |
| N / 14 | Physical environment |
| O / 15 | Formal care |
| P / 16 | Medication |
| Q / 17 | Global evaluation from the overall assessment |
| R / 18 | Responsibility and directives (administrative information on legal representative of patient. if any; availability of advance directive and living wills) |
