# Supplementary material for: Derivation of a frailty index from the resident assessment instrument – home care adapted for Switzerland: a study based on retrospective data analysis
Source: BMC Geriatr. 2017 Sep 7;17:205. doi: 10.1186/s12877-017-0604-3 (PMC5590146; doi:10.1186/s12877-017-0604-3)
Supplement: Supplementary file 2 — Set of items selected in the Swiss RAI-HC MDS to derive the FI. This supplementary information provides the set of items selected in the Swiss RAI-HC MDS to derive the FI. For each item, the information includes the original code from the reference manual, the health domain documented and the specific outcome assessed. (DOCX 19 kb) [file 12877_2017_604_MOESM2_ESM.docx]

**Additional file 2: Table S2.** Set of items selected in the Swiss RAI-HC MDS to derive the FI.

| **Item code** | **Health domain** | **Outcome assessed.** |
| --- | --- | --- |
| B2  B3a | attention | Global cognitive functioning  Distractibility |
| B1a  B1b | memory | Short term memory  Procedural memory |
| C1  C2 | language | Expression  Comprehension |
| B3b  E2a | orientation | Incoherent speech  Wandering |
| E1a  E1b  E1c  E1d  E1e  F4  K1d | emotion and affect | Negativity  Anger  Fears  Repeated complaints  Sadness  Lonelyness  Appetite loss |
| C3  D1 | sensory abilities | Hearing  Vision |
| H2a  H2b  H2c  H2e  H2f  H2g  H2h  H2i  H2j  H4  H5a  H5b  K6a  K6b | functional health | Mobility in bed  Transfer  Walk inside  Walk outside  Dress  Eat  Use the toilet  Self care  Bath  Climb strairs  Physical activity  Outing  Gait  Fear of falling |
| L2a  L2b  L3  BMI | nutrition | Fluid intake  Solid intake  Nutritional intake  Body Mass Index |
| P1 | medication | Number of different medications over the past 7 days |
| I1  I2  I3  K1b  K1c  K1g  K1h  K2  K3  L5  L6  M1  M4 | physiology | Bladder incontinence  Bowel incontinence  Incontinence device  Transit of solids  Transit of liquids  Appetite loss  Edema  Sleep disturbance  Dyspnea  Fatigue  Weight loss  Skin problems  Feet problems |
| K4a  K4b  P5e | pain | Frequent pain  Intense pain  Analgesics |
