# Supplementary material for: Derivation of a frailty index from the resident assessment instrument – home care adapted for Switzerland: a study based on retrospective data analysis
Source: BMC Geriatr. 2017 Sep 7;17:205. doi: 10.1186/s12877-017-0604-3 (PMC5590146; doi:10.1186/s12877-017-0604-3)
Supplement: Supplementary file 3 — Coding applied to the items selected in the Swiss RAI-HC MDS to derive the FI. This supplementary information reports the detailed coding applied to each of the items selected in the Swiss RAI-HC MDS to derive the FI. (DOCX 19 kb) [file 12877_2017_604_MOESM3_ESM.docx]

**Additional file 3: Table S1.** Coding applied to the items selected in the Swiss RAI-HC MDS to derive the FI.

| **MDS original variable type and item code** | | **Coded** |
| --- | --- | --- |
| *Original binary coding* | | |
|  | B1a, B1b, H5a, K6a, K6b, L2a, L2b, L5, L6, M1, M4, P5e | 0=absence ;  1=presence |
|  |  |  |
| *Original categorical coding (scoring the gradient of deficit)* | | |
|  | B2, K1b, K1c, K1d, K1g, K1h, K4b, L3, K3, K4a, H5b | 0=absence ;  1=presence irrespective of grading |
|  |  |  |
| *Original categorical coding with additional “does not answer” modality* | | |
|  | E1a, E1b, E1c, E1d, E1e, E2a, F4 | 0 = absence ;  1 = presence irrespective of grading and “does not answer” |
|  |  |  |
| *Original categorical coding with additional “newly observed” modality* | | |
|  | B3a, B3b | 0 = absence ;  1 = presence irrespective newly observed or not |
|  |  |  |
| *Original categorical coding with additional “most of the time absent” modality* | | |
|  | C1, C2, C3, D1, I1, I2, I3, K2 | 0 = totally absent ;  0.5 = most of the time absent;  1 = present in the remaining cases |
|  |  |  |
| *Original categorical coding with additional “with help” modality* | | |
|  | H2a, H2b, H2c, H2e, H2f, H2g, H2h, H2i, H2j, H4 | 0 = independent;  0.5 = with help not requiring strength;  1= with help requiring strength or dependent |
|  |  |  |
| *Original continuous value* | | |
|  | Body mass index (BMI) | 0 = 21 < BMI ≥ 30  1 = BMI < 21 or BMI ≥ 30 |
|  |  |  |
|  | Number of different medications over the past 7 days | 0 = absent, [0-2];  1 = marginally present, [3-8];  2 = present, > 8; |
